# Peripapillary Arterial Circle of Zinn-Haller: Location and Spatial Relationships with Myopia

**DOI:** 10.1371/journal.pone.0078867

**Published:** 2013-11-01

**Authors:** Jost B. Jonas, Leonard Holbach, Songhomitra Panda-Jonas

**Affiliations:** 1 Department of Ophthalmology, Medical Faculty Mannheim, Heidelberg University, Mannheim, Germany; 2 Department of Ophthalmology, Friedrich-Alexander University Erlangen-Nürnberg, Erlangen, Germany; University of Houston, United States of America

## Abstract

**Purpose:**

To measure histomorphometrically the location of the peripapillary arterial circle of Zinn-Haller (ZHAC) and assess its associations with axial length.

**Methods:**

Using a light microscope, we measured the distance from the ZHAC to the peripapillary ring (optic disc border), the merging point of the dura mater with the posterior sclera (“dura-sclera point”), and the inner scleral surface. In the parapapillary region, we differentiated between beta zone (presence of Bruch's membrane, absence of retinal pigment epithelium) and gamma zone (absence of Bruch's membrane). The peripapillary scleral flange as roof of the orbital cerebrospinal fluid space was the connection between the end of the lamina cribrosa and the posterior full-thickness sclera starting at the dura-sclera point.

**Results:**

The study included 101 human globes (101 patients) with a mean axial length of 26.7±3.7 mm (range: 20.0–39.0 mm). The distance between the ZHAC and the peripapillary ring increased significantly with longer axial length (*P*<0.001; correlation coefficient r = 0.49), longer parapapillary gamma zone (*P*<0.001;r = 0.85), longer (*P*<0.001;r = 0.73) and thinner (*P*<0.001;r = −0.45) peripapillary scleral flange, and thinner sclera posterior to the equator (*P*<0.001). ZHAC distance to the peripapillary ring was not significantly associated with length of parapapillary beta zone (*P* = 0.33). Including only non-highly myopic eyes (axial length <26.5 mm), the ZHAC distance to the disc border was not related with axial length (*P* = 0.84). In non-highly myopic eyes, the ZHAC was located close to the dura-sclera point. With increasing axial length and decreasing thickness of the peripapillary scleral flange, the ZHAC was located closer to the inner scleral surface.

**Conclusions:**

The distance between the ZHAC and the optic disc border is markedly enlarged in highly myopic eyes. Since the ZHAC is the main arterial source for the lamina cribrosa blood supply, the finding may be of interest for the pathogenesis of the increased glaucoma susceptibility in highly myopic eyes.

## Introduction

The intralaminar region of the optic nerve head is a major bottleneck and a locus minoris resistenciae of the whole visual afferent pathway. Nowhere else are the retinal ganglion cell axons or optic nerve fibers so densely packed and simultaneously have to pass through a pressure gradient such as between the intraocular space and the retrobulbar cerebrospinal fluid compartment [1]. This translaminar pressure difference changes periodically with the different time shifts of the ocular pressure pulse and the orbital cerebrospinal fluid pressure pulse [2]. The blood supply to the intralaminar optic nerve head region is therefore of high importance for the physiology and pathophysiology of the optic nerve head. According to the landmark studies by Hayreh and others, the peripapillary arterial circle of Zinn-Haller (ZHAC) gives off branches to the intralaminar region of the optic nerve head and has been considered to be the major arterial contributor to the vascular system in the lamina cribrosa [3]–[7]. The ZHAC is located within the posterior sclera, circling the optic nerve head. Described as early as 1754 and 1755 by Zinn and Haller [8], [9], previous studies examined the location of the ZHAC [10]–[23]. None of these investigations, however, was directed to assess the location of the ZHAC, and in particular its distance to the lamina cribrosa, in highly myopic eyes. According to Hagen-Poiseuille's law, the flow in a tube is directly proportional to its length, so that, despite restrictions in the application of Hagen-Poiseuille's law on blood flow, the distance between ZHAC and the lamina cribrosa may be of importance for the blood perfusion in the lamina cribrosa. We, therefore conducted this study to measure the location of ZHAC and its distance to the optic nerve head. We addressed in particular highly myopic eyes since previous hospital-based and population-based studies have revealed that high axial myopia is a major risk factor for glaucomatous optic neuropathy [24].

## Methods

### Ethics Statement

The study was approved by the ethics commission II of the Medical Faculty Mannheim of the Ruprecht-Karls-University Heidelberg, Germany. Since the globes had been enucleated up to 60 years ago, the ethics committee had decided that an informed consent, verbal or written, by the patients was not necessary to comply with the requirements of the principles expressed in the Declaration of Helsinki.

The histomorphometric study included human globes which either had been enucleated due to painful absolute glaucoma or which had been enucleated because of a malignant choroidal melanoma or which had been enucleated to complications of preceding surgeries or penetrating injuries. The characteristics of the study in general have already been described previously [25]. In the glaucomatous group, vision was completely or almost completely lost, and enucleation became necessary usually due to intractable pain which could not be treated by medication. At the time when the tumor eyes were enucleated, no other treatment modalities such as radiologic brachytherapy were available or were thought not to be suitable for tumor removal with respect to its location and size. Some of the globes have already been included in previous histomorphometric studies and have described in detail previously [1], [26]. Immediately after enucleation, the globes were fixed in a solution of 4% formaldehyde and 1% glutaraldehyde and processed for histological sectioning. The globes were prepared in a routine manner for light microscopy. An anterior-posterior segment going through the pupil and the optic nerve was cut out of the fixed globes. These segments were dehydrated in alcohol, imbedded in paraffin, sectioned for light microscopy, and stained by the Periodic-Acid-Shiff (PAS) method or by hematoxylin-eosin. For all eyes, one section running through the central part of the optic disc was selected for further evaluation. The thickness of the sections was 8 µm.

**Figure 1:**
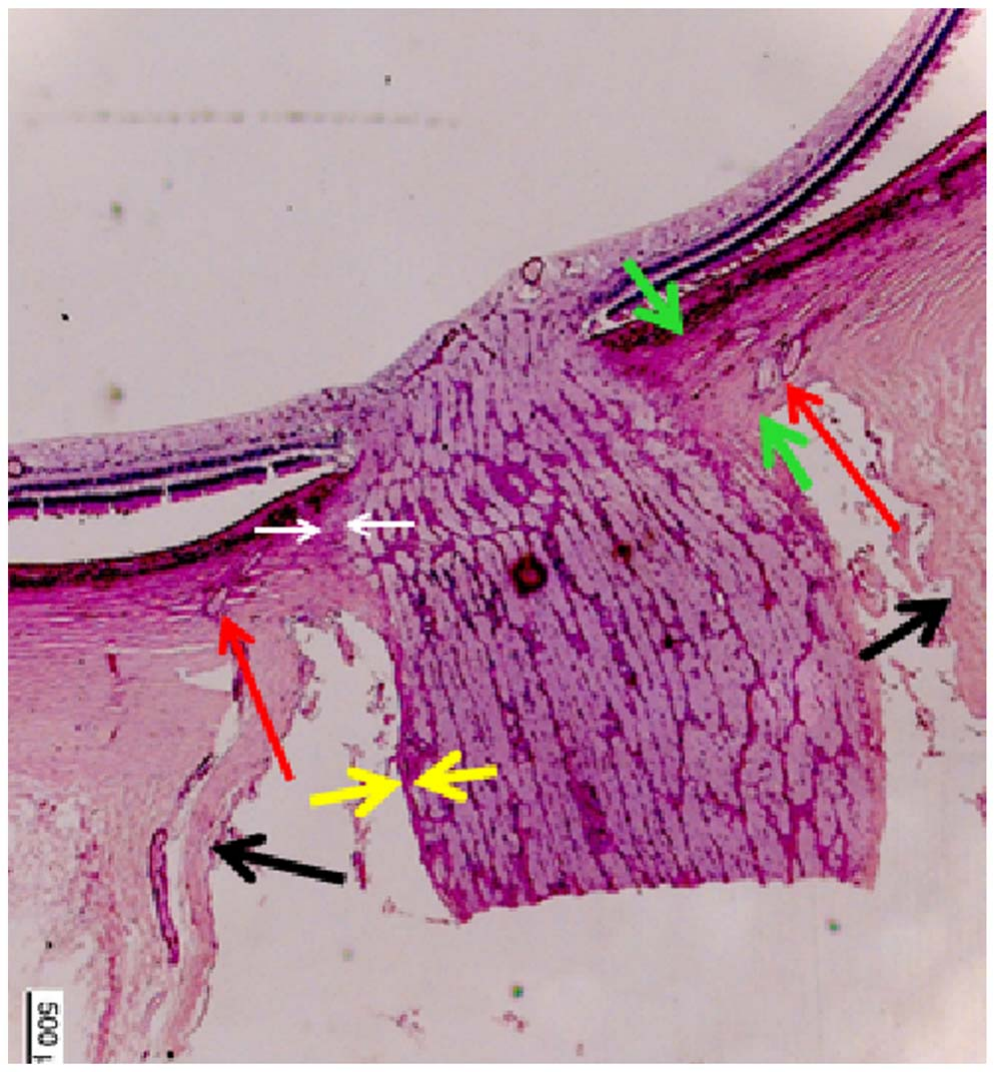
Histophotograph showing the optic nerve head of a non-highly myopic eyes with the peripapillary arterial circle of Zinn-Haller (red arrows), located at the merging point of the dura mater (black arrows) with the scleral at the end of the peripapillary scleral flange (between green arrows), the pia mater (yellow arrows), and the peripapillary ring (white arrows).

Using a scale in the ocular of the microscope, we measured the distance between the ZHAC and the merging point between the dura mater and the sclera (“dura-sclera point”), the distance between the ZHAC and the peripapillary ring as the continuation of the pia mater, and the distance between the ZHAC and the inner scleral surface (Fig. 1). We had additionally measured [27]:

- the axial length of the globes;
- the length of parapapillary beta zone defined as the region in which Bruch's membrane was not covered by retinal pigment epithelium cells;
- the length of parapapillary gamma zone defined as the parapapillary region without Bruch's membrane;
- the length of the peripapillary scleral flange defined as scleral bridge between the peripapillary ring as continuation of the pia mater of the optic nerve and the dura-sclera point; the peripapillary scleral flange formed the anterior roof of the orbital cerebrospinal fluid space;
- the thickness of the peripapillary scleral flange;
- the thickness of the sclera measured at the pars plana, ora serrata, equator, the midpoint between the equator and posterior pole, the peripapillary region just outside of the dura mater, and the posterior pole.
- the thickness of the choroid measured at the pars plana, ora serrata, equator, the midpoint between the equator and posterior pole, the peripapillary region just outside of the dura mater, and the posterior pole.

The axial length was measured using calipers after the globes were fixed in formaldehyde and glutaraldehyde, usually before opening and cutting the globes.

The statistical analysis was performed using a commercially available statistical software package (SPSS for Windows, version 21.0, SPSS Inc., Chicago, IL). In a first step of the statistical analysis, we calculated the means and standard deviations as well as medians and ranges. In a seconds step, univariate associations were tested between the parameters. The third step included multivariate analyses of these associations taking into account potentially confounding factors. All *P*-values were 2-sided and considered statistically significant when less than 0.05.

## Results

The study included 101 human globes (101 patients) with a mean age of 59.9±17.7 years (median: 61.5 years; range: 24–88 years) and a mean axial length of 26.7±3.7 mm (median: 26.0 mm; range: 20.0 – 39.0 mm). The study sample was differentiated into a non-highly myopic group without glaucoma (n = 23 globes), a non-highly myopic group with glaucoma (n = 30 globes), a highly myopic group without glaucoma (n = 12 globes), and a highly myopic group with glaucoma (n = 36 globes). High myopia was defined by an axial length of ≥26.5 mm. The ZHAC was detected in all eyes.

**Figure 2:**
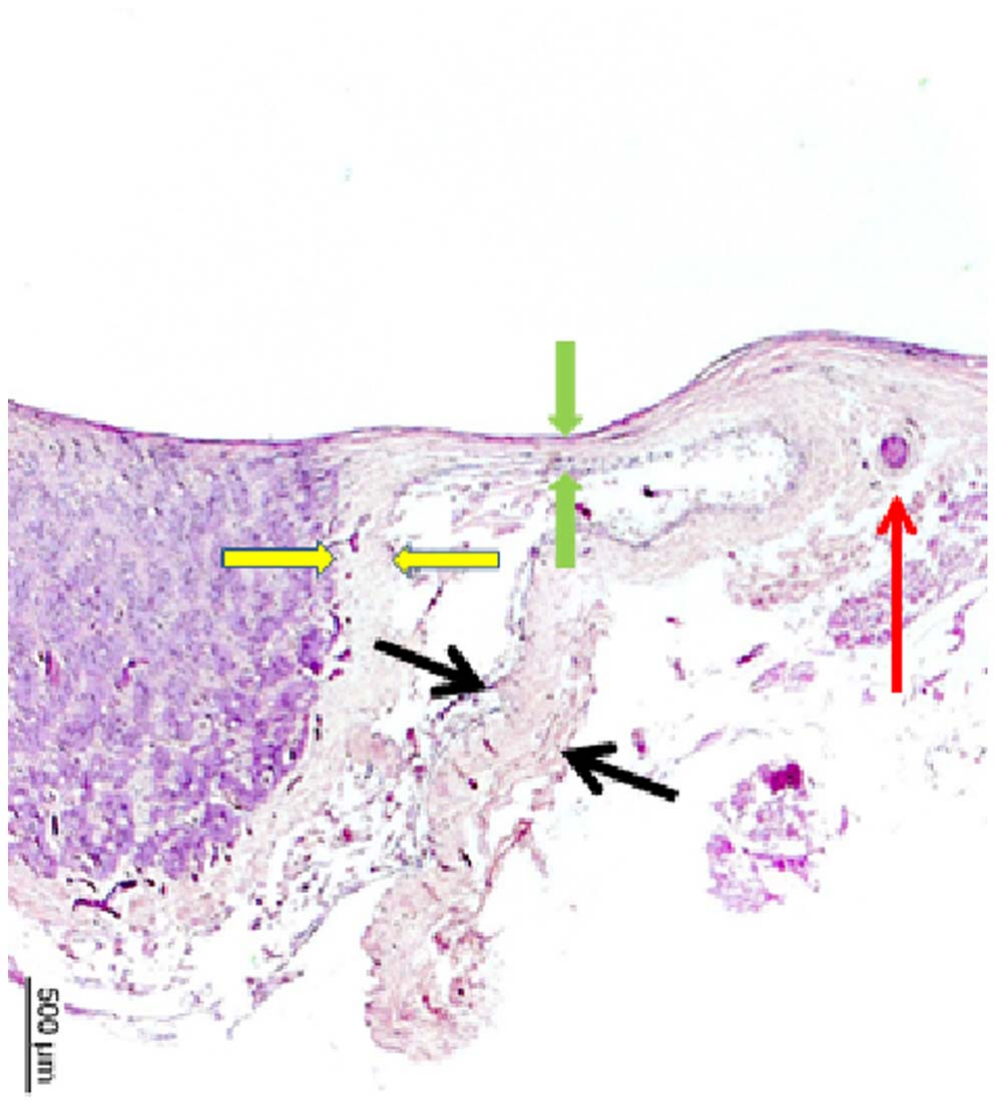
Histophotograph showing the optic nerve head of a highly myopic eyes with the peripapillary arterial circle of Zinn-Haller (red arrow), located the merging point of the dura mater (black arrows) with the scleral at the end of the peripapillary scleral flange (between green arrows), the pia mater and the peripapillary ring as the continuation of the pia mater (between yellow arrows).

In the non-highly myopic non-glaucomatous eyes, the mean distance between ZHAC and the peripapillary ring was 548±176 µm and ranged between 282 µm and 870 µm (Table 1). The ZHAC was located close to the dura-sclera point (Fig. 1, 2). Its distance to the inner scleral surface was 317±77 µm (Table 1). Within this non-highly myopic non-glaucomatous group, the distance ZHAC to peripapillary ring was not significantly associated with age (*P* = 0.10) nor axial length (*P* = 0.84).

**Table 1:** Histomorphometric Measurements in Enucleated Human Globes (Mean±Standard Deviations).

Parameter	Non-Highly Myopic Non-Glaucomatous Group	Non-Highly Myopic Glaucomatous Group	*P*-Value (1)	Highly Myopic Non-Glaucomatous Group	Highly Myopic Glaucomatous Group	*P*-Value (2)	*P*-Value (3)	
N	23	30		12	36			
Axial Length (mm)	23.5±1.2 (20.0 to 25.0)	24.0±1.4 (21.0 to 26.0)	0.18	29.5±2.2 (27.0 to 34.0)	30.0±2.9 (27.0 to 39.0)	0.56	<0.001	
Distance (µm) between ZHAC and:								
Peripapillary Ring	548±176 (282 to 870)	590±191 (212 to 1011)	0.41	930±434 (353 to 1645)	969±634 (329 to 2820)	0.81		<0.001
Merging Point between Dura Mater and Posterior Sclera (Negative values indicate a position between the dura-sclera point and the peripapillary ring)	6±59 (-118 to 235)	−45±94 (−282 to 118)	0.06	−198±243 (−705 to 0)	−157±472 (−1833 to 705)	0.70		0.03
Inner Scleral Surface	317±77 (176 to 470)	298±142 (129 to 776)2655	0.57	213±80 (106 to 376)	240±112 (94 to 541)	0.38		0.002

ZHAC: Peripapillary arterial circle of Zinn-Haller

*P*-Value (1): Statistical significance of the difference between the non-highly myopic non-glaucomatous group and the non-highly myopic glaucomatous group

*P*-Value (2): Statistical significance of the difference between highly myopic non-glaucomatous group and the highly myopic glaucomatous group

*P*-Value (3): Statistical significance of the difference between non-highly myopic groups and the highly myopic groups

The non-highly myopic non-glaucomatous group and the non-highly myopic glaucomatous group did not differ significantly in the distance between ZHAC and the peripapillary ring, dura mater, and the inner scleral surface, or in axial length (Table 1).

Within the highly myopic group, all measurements of the ZHAC did not differ significantly (all *P*>0.35) between the non-glaucomatous subgroup and the glaucomatous subgroup (Table 1).

Comparing the non-highly myopic groups with the highly myopic groups revealed significant differences for all ZHAC parameters. In the highly myopic eyes, the distance between ZHAC and the peripapillary ring was significantly (*P*<0.001) longer. In contrast to the non-highly myopic eyes in which the ZHAC was located close to the dura-sclera point (Fig. 1, 2), the highly myopic eyes showed a shifting of the ZHAC in direction to the optic nerve head.

**Figure 3:**
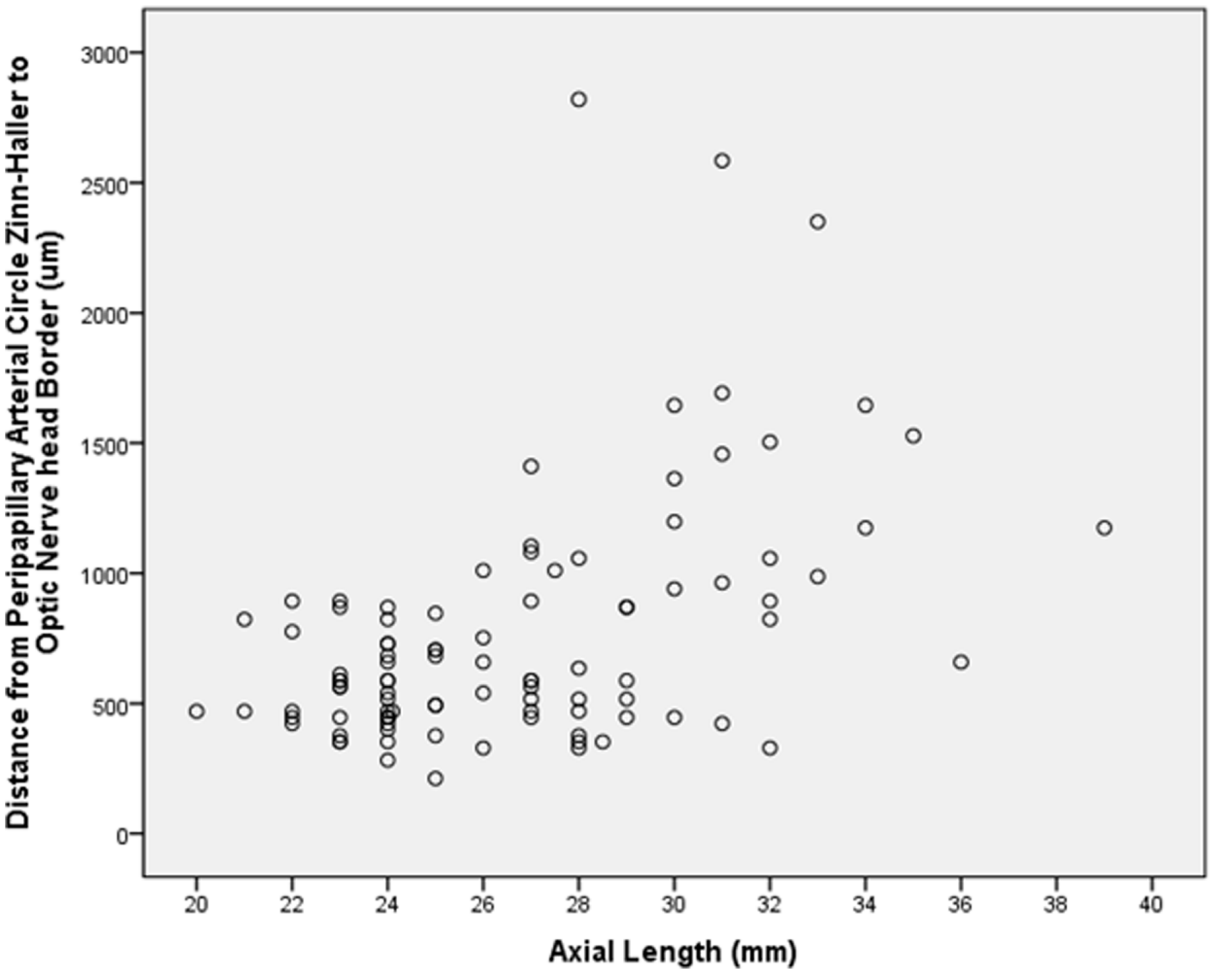
Graph showing the distribution of the distance between the peripapillary arterial circle of Zinn-Haller and the peripapillary ring in relation to axial length.

Taking the whole study sample, the distance between the ZHAC and the peripapillary ring increased significantly with longer axial length (*P*<0.001; correlation coefficient r = 0.49; equation of the regression line: ZHAC Distance (µm) = 61.5×Axial Length (mm) −876) (Fig. 3). In univariate analysis, the ZHAC distance to the peripapillary ring was additionally associated with a longer parapapillary gamma zone (*P*<0.001; r = 0.85), a longer peripapillary scleral flange (*P*<0.001; r = 0.73), a thinner peripapillary scleral flange (*P*<0.001; r = −0.45), a thinner sclera thickness at the midpoint between the equator and posterior pole (*P*<0.001), parapapillary region (*P*<0.001) and posterior pole (*P*<0.001), and a thinner choroid in the parapapillary region (*P* = 0.01) and at the posterior pole (*P* = 0.04). The ZHAC distance to the peripapillary ring was not significantly associated with the length of parapapillary beta zone (*P* = 0.33), and scleral thickness at the pars plana (*P* = 0.85), ora serrata (*P* = 0.28) and equator (*P* = 0.07).

A multivariate analysis included the ZHAC distance to the peripapillary ring as dependent variable and all variables as independent parameters which were significantly associated with the ZHAC distance in the univariate analysis. It revealed that the ZHAC distance to the peripapillary remained to be significantly associated with the length of the peripapillary scleral flange while all other parameters including axial length were no longer significantly associated with the ZHAC distance.

**Figure 4:**
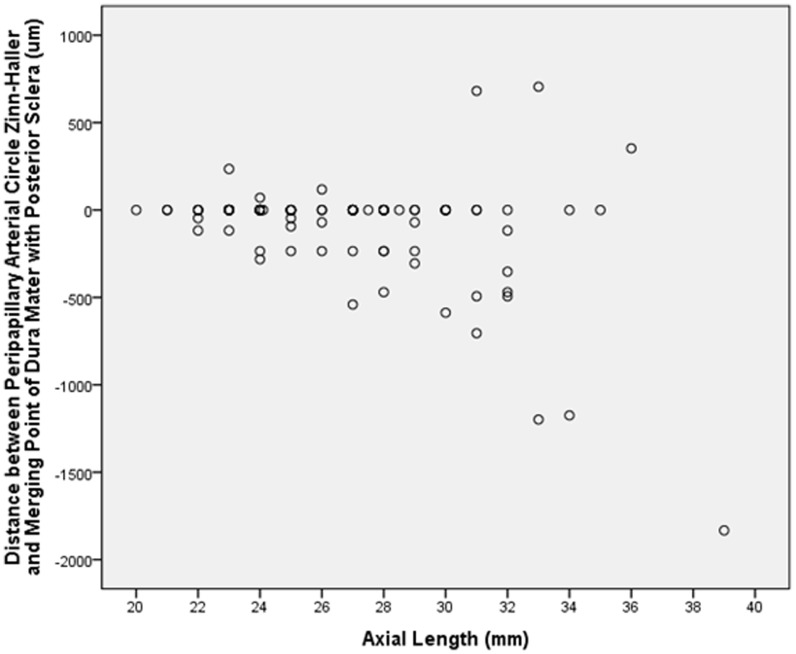
Graph showing the distribution of the distance between the peripapillary arterial circle of Zinn-Haller (ZHAC) and the merging point of the optic nerve dura mater with the posterior sclera in relation to axial length. Negative values mean location of the ZHAC between the merging point and the peripapillary ring; positive values mean ZHAC location peripheral to the merging point.

**Figure 5:**
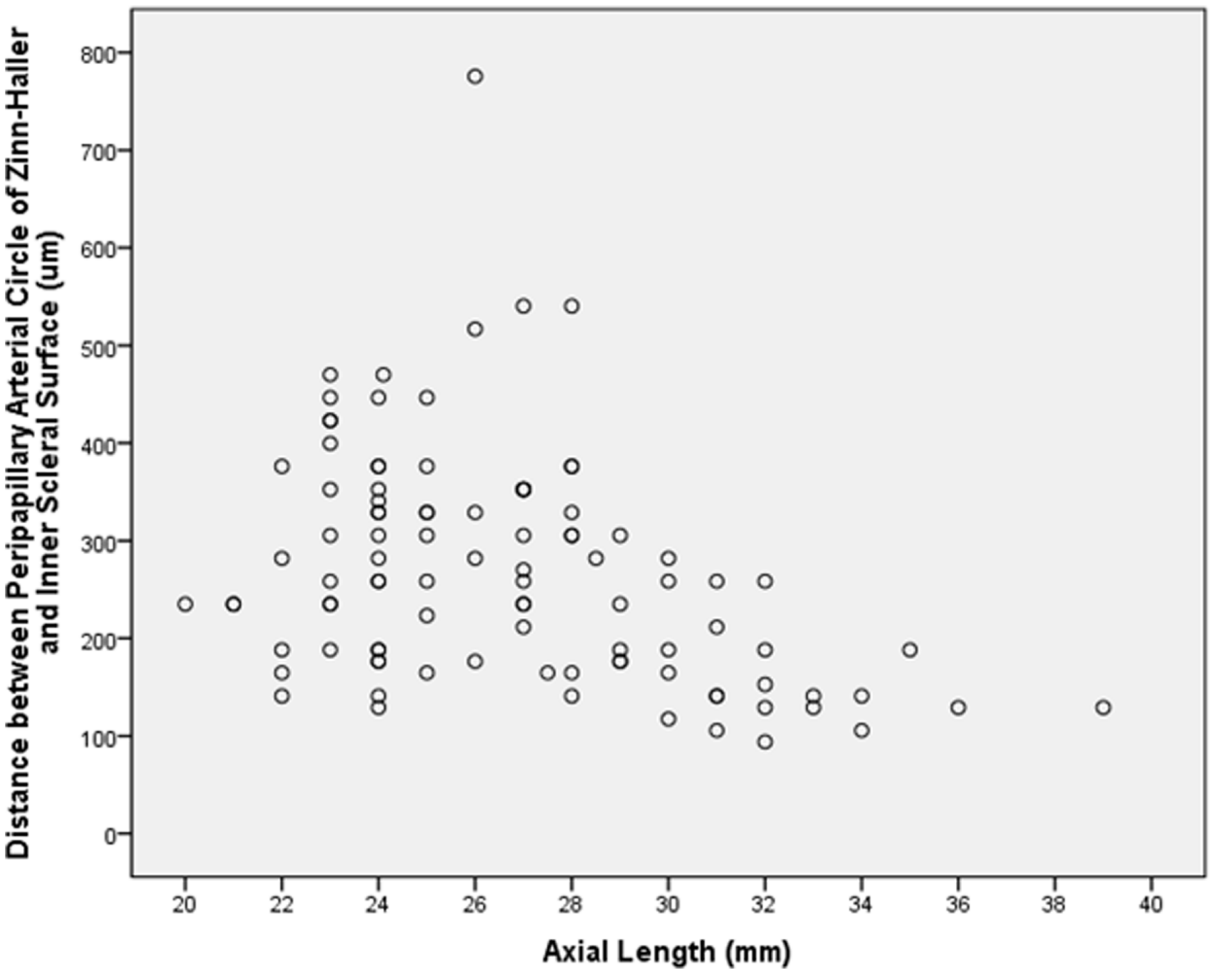
Graph showing the distribution of the distance between the peripapillary arterial circle of Zinn-Haller (ZHAC) and the inner scleral surface in relation to axial length.

The distance measurements between the ZHAC and the dura-sclera point decreased significantly with longer axial length (with negative values for a position between the dura-sclera point and the peripapillary ring), indicating a shifting of the ZHAC away from the dura-sclera point in direction to the optic nerve head (Fig. 4). The distance between the ZHAC and the inner scleral surface decreased with longer axial length indicating that the ZHAC was located closer to the inner scleral surface in axially elongated eyes (Fig. 5). In multivariate analysis, the ZHAC location in relation to the inner scleral surface was only associated with the thickness of the peripapillary scleral flange (*P*<0.001) while all other parameters including axial length were no longer significantly associated.

## Discussion

In our histomorphometric study on human globes the distance between the ZHAC and the peripapillary ring as marker of the optic disc border increased significantly with longer axial length (*P*<0.001) as well as with other parameters associated with axial elongation. These parameters included a longer parapapillary gamma zone (*P*<0.001), a longer and thinner peripapillary scleral flange (*P*<0.001), and a thinner sclera and choroid at the posterior pole (*P*<0.001). The relationship between a longer ZHAC distance to the optic disc border and axial length was valid only if the highly myopic group was included into the statistical analysis. In the non- highly myopic group, ZHAC distance to the optic disc border was not related with axial length. As the distance between the ZHAC and the disc border was not significantly related to glaucoma, it was neither correlated with the length of parapapillary beta zone. While in non-highly myopic eyes, the ZHAC was located close to dura-scleral point, the ZHAC shifted in direction to the optic nerve head in highly myopic eyes. In addition, its location got closer to the inner scleral surface as a function of the thinning of the peripapillary scleral flange.

The results of our study agree with the findings obtained in previous investigations. Ko and colleagues examined 42 human enucleated eyes and found that a distance between the ZHAC and the ONH border of 403±352 µm (0–1050 µm) [11], [12]. These data were rather similar or slightly smaller to those obtained in our study (548±176 µm). Reasons for the slight differences between Ko's results and our findings may be differences in the techniques applied (flat preparations, either fixed and stained with hematoxycilin/eosin or wet preparations as in Ko's study versus only fixed specimen in our study), and difference I the study samples including inter-ethnic differences. The measurements obtained in our study were also similar to those taken in a previous study by the similar group of researchers [25]. It may be noted that for the present study, all eyes were re-measured by a different examiner (JBJ) without knowledge of the previous measurements. Both studies also agree in that non-highly myopic glaucomatous eyes and non-highly myopic non-glaucomatous eyes did not differ in the location of the ZHAC. The findings of our histomorphometric study complement the recent clinical study by Ohno-Matsui and colleagues who imaged the ZHAC by indocyanine-green angiography and enhanced depth imaging of optical coherence tomography in 94 highly myopic eyes defined as an axial length of >26.5 mm or a myopic refractive error <−8 diopters [28]. Ohno-Matsui and coworkers found the ZHAC within the area of myopic conus, with the vessels appearing as a hyporeflective circle within the peripapillary sclera. The ZHAC usually had a rhomboid configuration.

The main finding of the present study was that the location of the ZHAC markedly changed in axially elongated (highly myopic) eyes: the distance of the ZHAC to the optic disc border markedly increased in strong relationship to the increase in axial length or the increase in the length of the peripapillary scleral flange in highly myopic eyes. Simultaneously, the ZHAC shifted from the dura-sclera point in direction to the optic nerve head border. It indicated that not only the distance to the lamina cribrosa increased for which the ZHAC is the main source of arterial blood supply, but also that the distance from the dura-scleral point to the ZHAC got larger. The ZHAC is fed by the short posterior ciliary arteries which run along the optic nerve dura mater. One may argue whether the increased distances may have an effect on the blood supply to the lamina cribrosa in highly myopic eyes, in particular since previous studies have shown an increased susceptibility for glaucomatous optic nerve damage in highly myopic eyes even if the intraocular pressure was normal [24].

The result of our study that the association between the ZHAC distance to the optic disc border and axial length was not found in non-highly myopic eyes agrees with the previous study in which mostly non-highly myopic eyes were included and in which the ZHAC location was not associated with axial length. The finding also agrees with previous clinical and hospital-based studies in that the increase in glaucomatous susceptibility with myopia was reported mainly for highly myopic eyes while eyes with minor or moderate myopia could usually did not show a markedly elevated glaucoma susceptibility as compared to emmetropic eyes [24].

The absence of a significant association between the ZHAC location and the length of parapapillary beta zone agrees with the absence of an association between the ZHAC location and glaucoma, since parapapillary beta zone, in contrast to parapapillary gamma zone, is related with glaucoma but not with axial length [27].

High myopia was defined by an axial length of ≥26.5 mm in our study what approximately is the equivalent of a refractive error of −8 diopters. In other studies, high myopia was considered to be a myopic refractive error of >−6 diopters as equivalent to an axial length of 25.5 to 26 mm. We defined high myopia by an axial length of ≥26.5 mm in this study as well as in previous investigations [24], [27], since we aimed to differentiate between medium myopic eyes without myopia associated stretching of the posterior pole and highly myopic eyes with myopic posterior stretching or enlargement of the posterior fundus. Hospital-based studies and population-based investigations had shown that a myopia associated enlargement of the optic disc and of the parapapillary atrophy, an increase in the prevalence of myopic retinopathy, and myopia associated thinning of the posterior sclera usually started at an axial length 26.5 mm [29]–[33].

There was a wide range in the measurements of the distance between the ZHAC and the disc border. To cite an example, in the non-highly myopic group without glaucoma, the measurements ranged from 282 µm to 870 µm. The reasons for this variability in the values may be first that any biologic quantitative parameters such as body height, optic disc site, and also the distance between the ZHAC and the disc border usually show a marked inter-individual variability. This variability may be even more marked in the arrangement of blood vessels. Second, variations in the histologic preparation of the slides and other technical parameters may have played a role.

Potential limitations of our study should be mentioned. First, due to postmortem swelling of the tissue after enucleation and due to the histological preparation of the slides, the measurements given in this study do not represent dimensions as determined in vivo. Since however, the swelling and preparation induced changes in the dimensions may have been valid both, myopic as hyperopic eyes, the conclusions of the study may not have been affected by this study limitation. Second, axial length was measured using calipers, so that the measurement included retinal, choroidal and scleral thickness at the posterior pole. This was different from the usual clinical measurement of axial length as linear distance from the anterior apex of the cornea to the anterior surface of the opposite retina. Also, the measurement was influenced by the fixation induced shrinkage of the globe, since the axial length of the globes was measured after fixation, usually before opening and cutting the globes. Third, some of the histologic slides did not run exactly through the optic disc center, but some slides were cut at an angle, rather than straight through the optic nerve head. This could have affected the distance measurements between the ZHAC and the disc border. Since however, this limitation of the study was true for the hyperopic eyes as well as the highly myopic eyes, it may have resulted in a higher noise in the measurements, but not have profoundly influenced the conclusions of the study, i.e. that the distance between the ZHAC and the optic disc border is markedly enlarged in highly myopic eyes. Fourth, according to a previous study, ZHAC in highly myopic eyes usually has a rhomboid shape suggesting that the distance between the ZHAC and the optic disc margin might be different between horizontal and vertical sections [28]. The majority of the globes of our study sample, in the highly myopic group as well as in the non-highly myopic group, were however, cut in a horizontal direction. Fifth, the study did not include normal human eyes as a control group but eyes which were enucleated either due to malignant choroidal melanomas or due to end-stage glaucoma. It is, therefore, not clear whether the results of our study can be generally transferred onto normal human eyes. Sixth, it was a laboratory-based retrospective study with a risk of a bias due to the selection of patients and eyes. Correspondingly, the mean axial length in our study was 26.7 mm, which was in the high myopia range. The reason was that the study samples was not population-based chosen but that the selection of globes into the study depended on the availability of enucleated globes. Seventh, one may argue whether the highly myopic eyes in our study sample had classic axial high myopia. One may discuss that the axial elongation was likely an effect of the underlying absolute glaucoma or malignant melanoma, so that the results could not be considered in terms of traditional axial myopia. Contradicting this assumption are the findings that the measurements of the ZHAC did not differ significantly between the non-glaucomatous highly myopic subgroup and the glaucomatous highly myopic subgroup (Table 1). In addition, population-based studies have not shown an association between axial high myopia or the prevalence of myopic retinopathy and intraocular pressure [34], [35], fitting with the notion that elevated intraocular pressure leads to an enlargement of the globes only in infants up to an age of approximately 2 to 3 years [36].

In conclusion, the distance between the ZHAC and the optic disc border is markedly enlarged in highly myopic eyes. Since the ZHAC is the main arterial source for the lamina cribrosa blood supply, the finding may be of interest for the pathogenesis of the increased glaucoma susceptibility in highly myopic eyes.
